# Hydrogen-Rich Saline Inhibits Lipopolysaccharide-Induced Acute Lung Injury and Endothelial Dysfunction by Regulating Autophagy through mTOR/TFEB Signaling Pathway

**DOI:** 10.1155/2020/9121894

**Published:** 2020-01-30

**Authors:** Zhiling Fu, Ze Zhang, Xiuying Wu, Jin Zhang

**Affiliations:** ^1^Department of Anesthesiology, Shengjing Hospital of China Medical University, No. 36 Sanhao Street, Shenyang, Liaoning Province 110004, China; ^2^Department of Orthopedics, Shenyang 739 Hospital, No. 121 Yellow River North Street, Huanggu District, Shenyang, Liaoning Province 110004, China

## Abstract

**Background:**

Hydrogen-rich saline (HRS) has strong anti-inflammatory, antioxidative stress, and antiapoptotic properties. The study focused on the protection of HRS on lipopolysaccharide (LPS)-induced acute lung injury (ALI) in rat models and the relationship with autophagic regulation and mTOR/TFEB signaling pathway. *Material and Methods*. The LPS-induced ALI rats' model was established. Pathohistological change in lung tissue was detected by hematoxylin-eosin staining. The inflammatory cytokines were examined by enzyme-linked immunosorbent assay (ELISA). The key apoptosis proteins and autophagy-relevant proteins were analyzed by western blotting. In vitro, HPMEC models of ALI were treated with LPS. The inflammatory cytokines were detected. Apoptosis rate was determined by flow cytometry. The autophagy and mTOR/TFEB signaling pathway-related proteins were detected by western blot and immunohistochemical staining.

**Results:**

HRS attenuated LPS-induced ALI and apoptosis both in *vivo* and in *vitro*. HRS attenuated inflammatory response, inhibited apoptosis, induced and activated autophagy in LPS-induced ALI model, and downregulated mTOR/TFEB signaling pathway. The protection of HRS can be blocked by autophagy inhibitor. Moreover, mTOR activator reversed HRS protection and mTOR inhibitor enhanced HRS protection in LPS-induced model and HRS activated autophagy via mTOR/TFEB signaling pathway.

**Conclusion:**

The results confirmed the protection of HRS in LPS-induced ALI by regulating apoptosis through inhibiting the mTOR/TFEB signaling pathway.

## 1. Introduction

ALI and acute respiratory distress syndrome (ARDS) are major complications in severe sepsis, increasing the risk of the development of multiple organ failure [1, 2]. ALI was estimated to lead to nearly 75,000 people dying each year in the United States [3]. Because of its complex pathogenesis, ALI has no effective treatment [4]. Lipopolysaccharides (LPS), also known as lipoglycans and endotoxin, are large molecules constructing the outer membrane of Gram-negative bacteria [5, 6]. LPS can cause severe pneumonia and sepsis and activate the downstream nuclear factor-kappa B (NF-*κ*B) signaling pathway producing inflammatory mediators [7, 8]. LPS was widely used in preparation of ALI animal models [9–12]. Among pulmonary cells, vascular endothelial cells (ECs) perform the important barrier between intravascular (IV) and extravascular (EV), which are always the primary target of inflammatory agents in local or systemic inflammation. LPS, targeting ECs, lead to barrier dysfunction through promoting ECs cytoskeletal contraction, disrupting ECs junctions and reducing ECs [13]. As there is no effective treatment for LPS-induced pulmonary endothelial barrier dysfunction, the therapy of ALI focuses on killing bacteria and reducing organ damage caused by an inflammatory response.

Autophagy, or self-eating, is a conservative process that degrades and endocytoses dysfunctional proteins and organelles into autophagic vesicles [14]. The autophagy is an important homoeostatic mechanism to maintain normal cellular function [15]. LPS can stimulate autophagy in lung often accompanied by insufficient autophagy, leading to the accumulation of autophagosomes playing a harmful role in ALI [16].

Molecular hydrogen is a nonmetallic, colorless, tasteless, odorless, and nontoxic gas. Hydrogen-rich saline (HRS) is a beneficial antioxidant, produced by dissolving molecular hydrogen gas into normal saline under high pressures. HRS is characterized by high hydrogen content, weakly alkaline property, negative potential, and small molecule water as well as safe, nontoxic, strongly anti-inflammatory, antioxidative stress, and antiapoptotic properties [17]. HRS was reported to relieve lung injury caused by a burn or hyperoxia [18, 19]. HRS also activates autophagy to perform function [20, 21]. However, the therapeutic effects of HRS in LPS-induced pulmonary endothelial injury and its mechanism remain poorly understood.

Mammalian target of rapamycin (mTOR)/transcription factor EB (TFEB) pathway was an important pathway in regulating cell cycle through TFEB-dependent regulation of autophagic flux [22]. Inhibition of mTOR activity can cause many TFEBs to dephosphorylate, activate, and enter the nucleus. The activated TFEBs can coordinate lysosomal expression and regulate targeting genes transcription, which is related to autophagosome formation and lysosome activity [23, 24]. Therefore, we try to prove that HRS can induce autophagy via downregulating the mTOR-TFEB signaling pathway in LPS-induced ALI.

## 2. Material and Methods

### 2.1. Animal and Grouping

Thirty Sprague-Dawley rats, weighing 260–280 g, were purchased from Laboratory Animal Center of China Medical University (CMU). The animal experimental protocol was approved by the Institutional Animal Care and Use Committee of CMU (IACUC No. 2016018R). Rats were randomly divided into ALI group, HRS group, HRS control (HRSC) group, and control group (*n* = 10). In the ALI group, the rats model induced by LPS was established. In the HRS group, rats were daily intraperitoneally (i.p.) injected with HRS 5 mL/kg for 3 days after the preparation of the ALI model [25]. In the HRSC group, rats without LPS administration were injected (i.p.) with HRS 5 mL/kg for 3 days. Rats in the control group were administered i.p. with some amount of saline.

### 2.2. Cell Cultivation

Human pulmonary vascular endothelial cells (HPMECs) were purchased from Shanghai Zhong Qiao Xin Zhou Biotechnology Co., Ltd., China. HPMECs were cultured in RPMI 1640 medium supplemented with 10% fetal bovine serum.

### 2.3. ALI Model Establishment and Sample Collection

Firstly, rats were fasted for 12 h and had free access to drink. LPS (5 mg/kg, cat. no. L8880, Solarbio, Beijing, China) was slowly injected into the trachea of each rat to induce ALI [26]. 24 h after administration, rats were anesthetized by 2% pentobarbital sodium and then sacrificed by bloodletting from the abdominal aorta. The serum was separated from the blood. One portion of lung tissue was fixed in 10% formaldehyde and the other part was preserved at −80°C.

In the in vitro study, HPMECs in the LPS group were added with LPS (100 *μ*g/mL) firstly, cultured at 37°C under 5% CO_2_ for 24 h, and then cells were harvested. After LPS treatment for 1 hour, cells in the HRS group were added with HRS 10 *μ*L/mL for further cultivation. In the HRSC group, HPMECs without LPS treatment were added with HRS 10 *μ*L/mL and cultivated for 24 h. Then cells were collected.

### 2.4. HRS Preparation

According to a previous report [27], a 500 mL bottle was sealed with a rubber septum closure containing 200 mL saline, the air was exhausted, and then the bottle was connected to a hydrogen generator. Ultrapure hydrogen gas, purity of 99.999%, was continuously infused by a syringe needle. The hydrogen was dissolved in saline at a pressure of 0.4 MPa. The concentration of hydrogen was measured by gas chromatography and maintained at 0.79 mmol/L. The HRS was used in one day.

### 2.5. Hematoxylin-Eosin Staining

After fixation in formaldehyde for 48 hours, the lung tissue was dehydrated, cleared, paraffin-embedded, sliced into 4 *μ*m-thick sections, dewaxed, rehydrated, stained with hematoxylin for 5 min, washed with PBS, differentiated with hydrochloric acid ethanol, stained with eosin for 1 min, and mounted with neutral resin. Finally, pathological change of lung tissue was observed under the microscope.

### 2.6. Immunohistochemical Staining

Lung tissue sections were dewaxed, rehydrated, treated with 3% H_2_O_2_ for 15 min, washed with PBS, and dissolved in 0.1 M sodium citrate solution for antigen retrieval. After treatment by TFEB antibody (ab2636, Abcam, USA) at 4°C overnight, slices were washed with PBS and incubated with biotin-labeled secondary antibody. After incubation at 37°C for 30 min, slices were washed with PBS, developed with 3,3′-diaminobenzidine and counterstained with hematoxylin, mounted with neutral gum, and observed under the microscope.

### 2.7. ELISA Assay

The levels of IL-1*β* (Cloud-Clone Corp, SEA563Ra, Wuhan, China), TNF-*α* (Cloud-Clone Corp, SEA133Ra), IL-10 (Cloud-Clone Corp, SEA056Ra), IL-6 (Cloud-Clone Corp, SEA079Ra) in serum and cell culture supernatant were detected according to the instructions.

### 2.8. MTT Assay

Approximately 8 × 10^4^ cells in logarithmic growth were seeded in 96-well plate. 20 *μ*L of MTT solution (5 mg/mL, cat. no. M8180, Solarbio) was added to each well, and then cells were cultured for 4–6 hours. After removal of the supernatant, 150 *μ*L of dimethyl sulfoxide was added and shaken for 10 min. The optical density of dimethyl sulfoxide was measured at 570 nm on a plate reader. The cell survival rate was calculated.

### 2.9. Detection of Cell Apoptosis by Flow Cytometry

Cells were collected and resuspended. Then cells were added with 5 *μ*L of Annexin V (BD Pharmingen, San Diego, CA, USA; 556547) and 5 *μ*L of propidium iodide (PI; BD Pharmingen; 556547) and incubated for 30 min in darkness. Then cells were washed by PBS and resuspended in 300 mL PBS. Cell apoptosis rate was calculated using Flow Jo software.

### 2.10. Western Blot Analysis

Cells or lung tissues were collected and homogenized and lysed by lysis buffer containing protease inhibitor (Thermo Fisher Scientific, Inc., Waltham, MA, USA; 89900) for 30 min on ice. The supernatant was collected. Protein concentration was determined using the BCA protein assay kit (Thermo Fisher Scientific, Inc.). Proteins were separated by SDS-PAGE and transferred on PVDF membrane. After addition of Bax (Abcam, ab32503), Bcl-2 (Abcam, ab59348), Caspase-3 (Abcam, ab13847), LC3A/B (CST, #4108), Beclin-1 (Abcam, ab62557), P62 (Abcam, ab56416), mTOR (Abcam, ab2732), p-mTOR (Abcam, ab137133), S6 (Abcam, ab40820), p-S6 (Abcam, ab12864), and TFEB (Abcam, ab174745) antibodies, PVDF membrane was incubated at 4°C overnight. After washing with PBS, the PVDF membrane was added with a horseradish peroxidase-labeled secondary antibody and incubated at room temperature for 2 h. Protein bands were visualized by ECL chemiluminescence detection kit and gel imaging system. The results were analyzed by Image Tools.

### 2.11. qRT-PCR

Total RNA from cells and lung tissue was extracted using Trizol reagent (Invitrogen, Carlsbad, CA, USA; 15596026) and reverse-transcribed into cDNA (Thermo, K1622). According to qRT-PCR kit instruction (Qiagen, Germany), mRNA expression of Bax, Bcl-2, Caspase-3, LC3B, Beclin-1, and P62 was measured. PCR was performed according to kit instructions. Primers sequences were shown in Table 1, synthesized by Sangon Biotech (Shanghai) Co., Ltd., China. PCR conditions were predenaturation at 95°C for 30 s followed by 40 cycles of denaturation at 95°C for 5 s and annealing at 60°C for 20 s. A melting curve was as follows: 95°C for 1 s, 65°C for 15 s, and 95°C for 5 s. After PCR, the amplification curves and melting curves were confirmed.

### 2.12. Statistical Analysis

All data were statistically analyzed using SPSS 19.0 software and expressed as the mean ± SD. *T*-test was used for pairwise comparison. One-way analysis of variance (ANOVA) with Tukey's multiple comparison post hoc test was used for comparison between groups. *P* < 0.05 was considered statistically significant.

## 3. Results

### 3.1. HRS Attenuated LPS-Induced ALI in Rat Models

**Figure 1:**
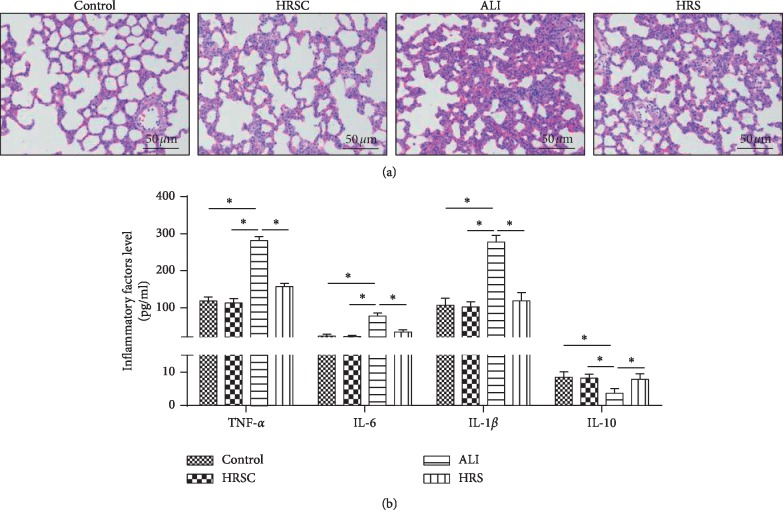
HRS attenuated LPS-induced ALI in rat models. (a) Hematoxylin-eosin staining (Scale bar = 50 *μ*m). (b) ELISA assay was used to detected inflammatory factor levels; the number of objects analyzed of rats in rats group is 10. Data were expressed as mean ± SD. Statistically significant differences: ^*∗*^indicates *P* < 0.05. ALI: acute lung injury; HRS: Hydrogen-rich saline. All data were analyzed by one‐way analysis of variance (ANOVA) with Tukey's multiple comparison post hoc test.

Firstly, comparing the control group with the HRS group, there is no significant change in lung tissue structure (Figure 1(a)); therefore HRS does not affect the normal rat lung function. Then Rat models of LPS-induced ALI were successfully established. HE staining showed lung tissue structural imperfection, alveolar wall thickening, pulmonary vascular engorgement, many inflammatory cells infiltration, and pulmonary endothelial cells necrosis. In the HRS group, less inflammatory cells were infiltrated and endothelial cell swelling was obviously attenuated (Figure 1(a)). ELISA showed that IL-1*β*, IL-6, and TNF-*α* in the ALI group were significantly more than those in the control group (*P* < 0.05), and IL-10 in the ALI group was significantly less than that in the control group (*P* < 0.05). IL-1*β*, IL-6, and TNF-*α* were significantly decreased and IL-10 was significantly increased in the HRS group, compared with that in the ALI group (*P* < 0.05) (Figure 1(b)). These data suggested that HRS attenuate LPS-induced ALI in rat models.

### 3.2. HRS Inhibits Apoptosis in ALI Rat Models

**Figure 2:**
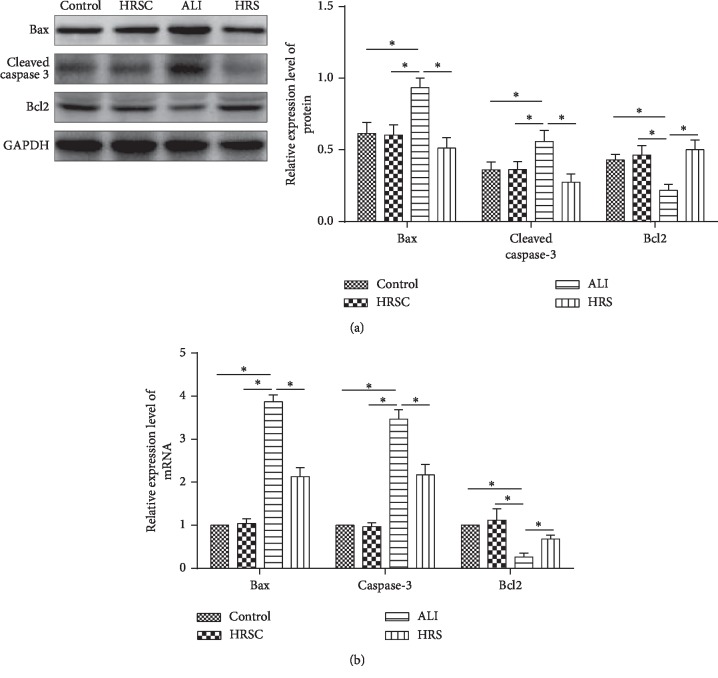
HRS attenuated ALI-induced apoptosis in rat models. Western blot assay and qRT-PCR were used to observe apoptosis-related protein and gene expression. (a) Western blot assay; (b) qRT-PCR assay; the number of objects analyzed of rats in rats group is 10. Data were expressed as mean ± SD. Statistically significant differences: ^*∗*^indicates *P* < 0.05 ALI: acute lung injury; HRS: Hydrogen-rich saline. All data were analyzed by one‐way analysis of variance (ANOVA) with Tukey's multiple comparison post hoc test.

Bax and Caspase-3 in the lung tissue were significantly increased and Bcl-2 was decreased in the ALI group, compared to the control group (*P* < 0.05) (Figure 2(a)). Bax and Caspase-3 were significantly decreased and Bcl-2 was significantly increased in the HRS group, compared to that in the ALI group (*P* < 0.05). These data were consistent with qRT-PCR results (Figure 2(b)). These results demonstrated that HRS inhibits apoptosis in ALI rat models.

### 3.3. HRS Attenuates LPS-Induced Inflammatory Response and Inhibited Apoptosis *In Vitro*

**Figure 3:**
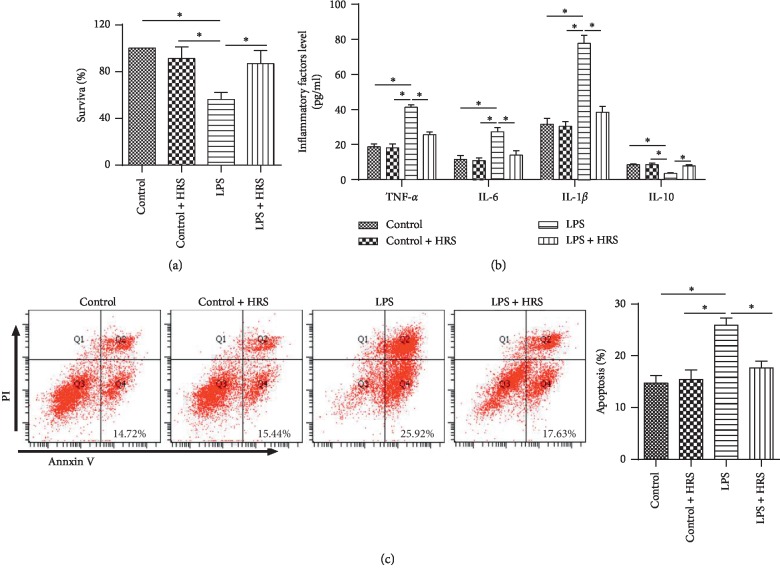
HRS attenuated LPS-induced inflammatory response and inhibited apoptosis. (a) Flow cytometry was used to detect the survival rate of HPMECs. (b) ELISA assay was used to detected inflammatory factor levels. (c) Flow cytometry was used to detect the apoptosis rate of HPMECs. All experiments of cells were repeated three times. Data were expressed as mean ± SD. Statistically significant differences: ^*∗*^indicates *P* < 0.05. LPS: lipopolysaccharide; HRS: Hydrogen-rich saline. All data were analyzed by one‐way analysis of variance (ANOVA) with Tukey's multiple comparison post hoc test.

We further validated the protection of HRS *in vitro*. Firstly, the results showed that there is no significant change in cell survival rate (Figure 3(a)) and apoptosis (Figure 3(c)) between the HRS group and the HRSC group, suggesting HRS has no effect on normal HPMECs cells. Moreover, the survival rate of HPMECs was significantly increased in the LPS + HRS group compared with cells in the LPS group (*P* < 0.05) (Figure 3(a)). The levels of IL-1*β*, IL-6, and TNF-*α* were significantly decreased, and IL-10 was significantly increased in the LPS + HRS group compared with that in the LPS group (all *P* < 0.05) (Figure 3(b)). In addition, flow cytometry showed that cell apoptosis was reduced in the HRS group compared with the LPS group (*P* < 0.05) (Figure 3(c)). These findings suggested that HRS attenuate LPS-induced inflammatory response in HPMECs and inhibit the apoptosis of HPMECs.

### 3.4. HRS Activates Autophagy in LPS-Induced ALI

**Figure 4:**
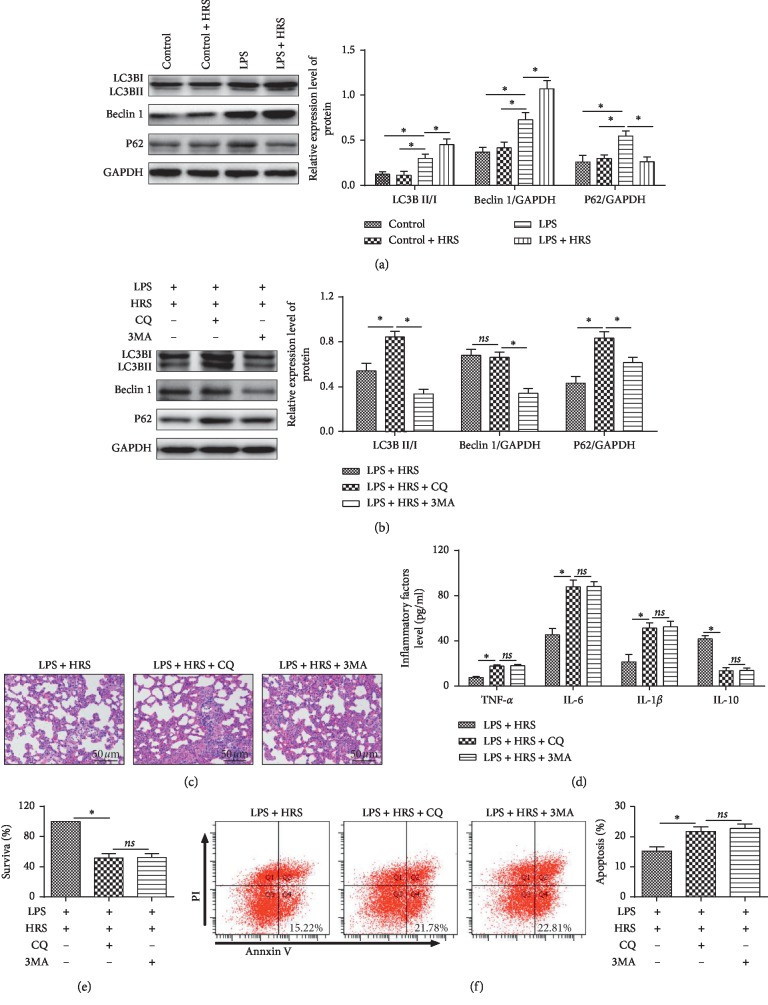
HRS activates autophagy in LPS induced ALI and inhibition of autophagy reverses the protective effect of HRS in LPS induced ALI. (a) and (b) Western blotting was used to observe the expression of LC3-II, P62, and Beclin-1 protein. (c) Hematoxylin-eosin staining (Scale bar = 50 *μ*m). (d) ELISA assay was used to detected inflammatory factor levels. (e) Flow cytometry was used to detect the survival rate of HPMECs. (f) Flow cytometry was used to detect the apoptosis rate of HPMECs. All experiments of cells were repeated three times. Data were expressed as mean ± SD. Statistically significant differences: ^*∗*^*P* < 0.05. LPS: lipopolysaccharide; HRS: Hydrogen-rich saline. CQ: chloroquine, an autophagy inhibitor; 3MA: 3-methyladenine, an autophagy inhibitor. All data were analyzed by one‐way analysis of variance (ANOVA) with Tukey's multiple comparison post hoc test.

To investigate the activation of autophagy in lung tissue under HRS treatment, Beclin-1, LC3-II, and P62 were detected by western blot. LC3-II, P62, and Beclin-1 were upregulated in the LPS group compared with the control group (*P* < 0.05). Moreover, P62 was downregulated in the LPS + HRS group, compared with the LPS group (Figure 4(a)). All these results revealed HRS could activate autophagy in LPS-induced ALI.

### 3.5. Inhibition of Autophagy Reverses HRS Protection in LPS-Induced ALI

To investigate whether the function of HRS depends on autophagy activation, we treated HPMEC or rats with autophagy inhibitor chloroquine (CQ) and 3MA. CQ could inhibit the process of a combination of the autophagosome with the lysosome, and 3MA can inhibit the autophagosome formation. Then autophagy, cell survival and apoptosis, lung injury, and inflammatory factors were detected in LPS + HRS, LPS + HRS + CQ, and LPS + HRS + 3MA groups. The results showed that Beclin-1 and LC3-II were lower and p62 was higher in the LPS + HRS + 3MA group compared to the LPS + HRS group. However, the change of Beclin-1 was not significant. And LC3-II and P62 were increased in the LPS + HRS + CQ group compared with the LPS + HRS group (Figure 4(b)). Compared with the LPS + HRS group, lung tissue injury aggravated in both the LPS + HRS + CQ and LPS + HRS + 3MA groups (Figure 4(c)), the expressions of IL-1*β*, IL-6, and TNF-*α* were increased (*P* < 0.05) the expression of IL-10 was decreased (*P* < 0.05) (Figure 4(d)), the survival rate of HPMECs was reduced (Figure 4(e)), apoptosis was increased (Figure 4(f)), and the protection of HRS was totally blocked.

### 3.6. HRS Activates Autophagy via Downregulating mTOR/TFEB Signaling Pathway

**Figure 5:**
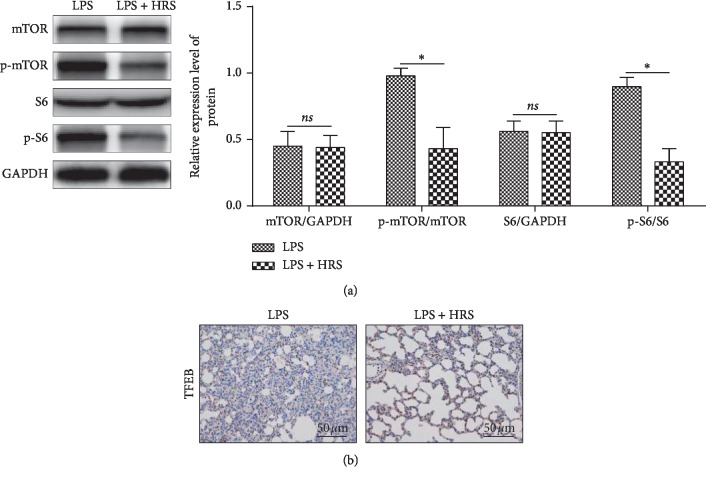
HRS activates autophagy via downregulating mTOR/TFEB signaling pathway. (a) Western blot assay was performed to detect the expression levels of p-mTOR and p-S6. (b) Immunohistochemical assay was performed to detect the expression of TFEB protein (Scale bar = 50 *μ*m). The number of objects analyzed of rats in rats group is 10. Data were expressed as mean ± SD. Statistically significant differences: ^*∗*^*P* < 0.05. LPS: lipopolysaccharide; HRS: Hydrogen-rich saline. All data were analyzed by *t*-test.

To further investigate the mechanism of HRS protection, we detected the expression of mTOR/TFEB-related proteins. Western blot results showed that HRS can significantly downregulate p-mTOR and p-S6 (Figure 5(a)), and nuclear TFEB was upregulated (Figure 5(b)). These results suggested that HRS may activate autophagy via downregulating the mTOR/TFEB signaling pathway.

### 3.7. HRS Protection in LPS-Induced ALI Was Dependent on mTOR/TFEB Signaling Pathway

**Figure 6:**
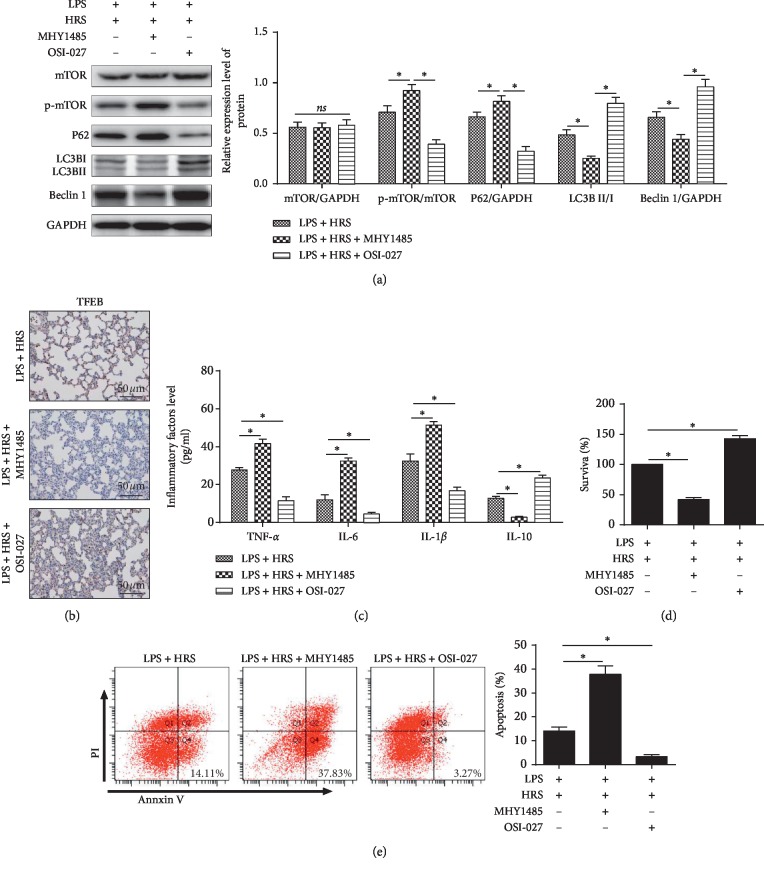
mTOR activator reversed HRS protection effect and mTOR Inhibitor enhanced HRS protection effect in LPS-induced injury. (a) Western blot assay was performed to detect the expression levels of p-mTOR, p-S6 LC3-II, P62, and Beclin-1 protein expression levels. (b) Immunohistochemical assay was performed to detect the expression of TFEB protein (Scale bar = 50 *μ*m). (c) ELISA assay was used to detect inflammatory factor levels. (d) Flow cytometry was used to detect the survival rate of HPMECs. (e) Flow cytometry was used to detect the apoptosis rate of HPMECs. All experiments of cells were repeated three times. Data were expressed as mean ± SD. Statistically significant differences: ^*∗*^indicates *P* < 0.05. LPS: lipopolysaccharide; HRS: Hydrogen-rich saline; MHY1485: an mTOR activator; OSI-027: an mTOR inhibitor. All data were analyzed by one‐way analysis of variance (ANOVA) with Tukey's multiple comparison post hoc test.

To further prove that the protection of HRS was regulated by inhibiting mTOR/TFEB pathway, mTOR activator MHY1485 and inhibitor OSI-027 were used to treat HPMEC, respectively. In the LPS + HRS + MHY1485 group, phosphorylated mTOR and P62 were increased, LC3-II and Beclin-1 were reduced (Figure 6(a)), nuclear TFEB proteins were downregulated (Figure 6(b)), IL-1*β*, IL-6, and TNF-*α* were increased, IL-10 was decreased (Figure 6(c)), the survival rate of HPMECs was reduced (Figure 6(d)), and apoptosis was increased (Figure 6(e)) compared with the LPS + HRS group. In the LPS + HRS + OSI-027 group, phosphorylated mTOR and P62 were decreased, LC3-II and Beclin-1 were increased (Figure 6(a)), nuclear TFEB proteins were upregulated (Figure 6(b)); IL-1*β*, IL-6, and TNF-*α* were decreased, IL-10 was increased (Figure 6(c)), the survival rate of HPMECs was increased (Figure 6(d)), and apoptosis was decreased (Figure 6(e)) compared with LPS + HRS group. These results suggested that mTOR activator reversed HRS protection and mTOR inhibitor enhanced HRS protection in ALI induced by LPS.

## 4. Discussion

Hydrogen is one of the simplest elements in nature. Gaseous Hydrogen (H_2_) is a colorless, odorless, tasteless, low-reducing diatomic gas. In 1975, Dole et al. reported in Science that 97.5% hydrogen was administrated with rats through breathing, effectively treating animal skin malignant tumors by the antioxidant effect of hydrogen. Hydrogen can quickly spread to cell membranes and penetrate the blood-brain barrier easily. Ohsawa et al. reported that H_2_ acts as a therapeutic and preventive antioxidant through reducing the strong oxidants such as hydroxyl radicals (·OH) and peroxynitrite (ONOO-) in cells. HRS has received extensive attention in past decades, having antioxidation, antiapoptosis, and anti-inflammatory effects. In our previous studies, we found that HRS attenuated endotoxin-induced lung dysfunction [28]. HRS treatment can alleviate LPS impaired lung function and upregulate the expression of AQP1 and AQP5 in rat's lung, inhibiting activation of p38 mitogen-activated protein kinase and Jun N-terminal kinase caused by LPS [29]. However, whether HRS can induce autophagy in LPS-induced ALI has not been reported.

Our study demonstrated that LPS can induce autophagy in rats' lung tissue and endothelial cell, and the elicited autophagy accompanied by insufficient completion of autophagy leading to the accumulation of autophagosomes. Autophagosomes were increased, and the degradation was incomplete; moreover the expressions of LC3-II, Beclin-1, and P62 were upregulated. HRS treatment could attenuate the inflammatory response caused by ALI, inhibiting apoptosis in injured lung tissue and cell cultures, activating autophagy and promoting autophagosomes clearance in injured lung tissue and cell model.

In pulmonary diseases, autophagic markers were elevated; therefore autophagy may initially act as a protective response to cell death [30]. Cardiovascular risk factors, including LPS, smoking, and ROS can induce endothelial autophagy [31, 32]. And the autophagic response can enhance endothelial survival under stress circumstances. Transplantation of mesenchymal stem cells can alleviate LPS-ALI through downregulation of miR-142a-5p and increase Beclin-1-mediated cell autophagy in pulmonary endothelial cells [33]. Autophagy can protect from ischemia/reperfusion-induced lung injury by alleviating blood-air barrier damage through ERK1/2 [34].

The process of autophagy includes the induction and formation of autophagosome and autophagosome-lysosome fusion. As autophagy occurs, autophagosomes are increased, LC3-II and Beclin-1 are upregulated, and P62 is downregulated accompanied by the degradation of autophagosome [35–37]. The abundance of autophagosomes may be due to increased autophagic activity or the accumulation of autophagosomes because of blocking autophagosome-lysosome fusion [38, 39]. Insufficient completion of autophagy can lead to exhaustion of autophagy and may be harmful to the body for the excess cumulation of autophagosomes as accumulated autophagosomes are extruded out of cells, leading to a proinflammatory response [16, 40, 41]. Some studies showed that autophagy seems to be harmful in LPS treated endothelial cells [42] due to the incompletion of autophagy, which was in accordance with our results.

Autophagy was regulated by the mTOR pathway and non-mTOR pathway, in which mTOR pathway plays a negative function in regulating autophagy. The inhibition of mTOR can enhance autophagy [14], leading to TFEB activation by dephosphorylation and translocation into the nucleus. Many lysosomal and autophagy proteins could promote transcription, and the TFEB entering the nucleus could promote cellular autophagy [23, 24]. TFEB regulates the transcription of lysosomal function and structure related proteins, including lysosomal membrane proteins, hydrolases, and the V-ATPase complex [43]. In cardiovascular disease, TFEB can protect the endothelial cell from inflammation and treat atherosclerosis [23]. Moreover, TFEB can promote autophagosome-lysosomal fusion; it has the potential to resolve autophagic accumulation and maintain the degradation pathway [44]. Our data suggested that HRS could activate autophagy by suppression of mTOR and promoting TFEB nuclear translocation, and TFEB activation can enhance lysosomal function, thus promoting autophagosome-lysosomal fusion to accelerate autophagosome degradation. MTOR can regulate the effect of HRS through regulating autophagy.

Taken together, our results proved that HRS attenuates the inflammatory response caused by LPS, inhibits apoptosis, activates autophagy, and regulates the ratio of p-mTOR and TFEB. These results confirmed that HRS exhibits protection in LPS-induced ALI through the mTOR/TFEB signaling pathway.

TFEB is one of the important proteins in the regulation of autophagy. TFEB can activate the transcription of many autophagy related proteins. Moreover, after incorporating into the nucleus, TFEB could enhance the autophagy activity and upregulate the expression of lysosomal pathway-related proteins, including the lysosomal membrane molecule LAMP1, then promoting lysosome release. TFEB is recognized as a key molecule for treating metabolic cumulative disease. TFEB overexpression can alleviate the accumulation of autophagosomes, mitochondrial division, and myocardial cell death in heart failure [45].

Studies have shown that mTORC1 activation negatively regulates autophagy by phosphorylating TFEB and inhibits its nuclear translocation, while mTORC1 inhibits TFEB-mediated nuclear translocation, which is the integration and coordination of energy degradation and recycle system [46]. MTORC1 is the key molecular in the balance of cellular stress and energy metabolism [47].

Our study showed that LPS inhibited the phosphorylation of mTOR in HPMEC cells and upregulated the expression of autophagy proteins, LC3-II and Beclin-1. The addition of saturated hydrogen saline inhibited the inflammatory response of ALI in rats, enhanced the autophagy level, upregulated LC3-II and Beclin-1, reduced P62, and promoted the degradation of autophagosomes of HPMEC cells. When mTORC1 agonist was added to HPMEC cells, the regulatory effect of HRS was inhibited, the expression of p-mTOR, p-S6, and TFEB was increased, while the effect of mTORC1 inhibitor was similar to that of HRS. These data indicate that this protective effect of HRS is most likely accomplished by the mTOR/TFEB signaling pathway. Current studies have confirmed that mTOR and autophagy are involved in the pathogenesis and development of ALI, but the detailed mechanism of its regulation of inflammatory response has not been deeply explored.

Our study did have a few limitations. First, we did not observe dynamic changes in autophagy over time. Instead, we compared the changes in autophagy levels in different intervention groups at 24 h after intervention. Also, except autophagy, HRS has been proved to participate in the protection of inflammation of LPS-induced ALI by mechanisms which were mentioned above.

## 5. Conclusions

HRS reduces the inflammatory response in ALI, inhibits the tissue and cell apoptosis response, activates tissue and autophagy, and alters the expression of p-mTOR and TFEB. These results demonstrate the protection of HRS in LPS-induced ALI by inhibiting the mTOR/TFEB signaling pathway.

## Figures and Tables

**Table 1:** qRT-PCR using gene primers.

Gene	Primer (5′⟶3′)
Bax	Forward: GACACTGGACTTCCTCCGG
	Reverse: GATTGCTGATGTGGATAC
Bcl-2	Forward: GGCTACGAGTGGGATACT
	Reverse: ACACGGCTGCCAGGACGT
Caspase-3	Forward: ACCACTGGATTTTCTGG
	Reverse: GCCCAAATAGAGGAGGCT
GAPDH	Forward: GTCATCAACGGGAAACC
	Reverse: CATGGAGAAGGCTGGGG

## Data Availability

The datasets used and/or analyzed during the current study are available from the corresponding author upon reasonable request.
